# Association of Human Intestinal Microbiota with Lifestyle Activity, Adiposity, and Metabolic Profiles in Thai Children with Obesity

**DOI:** 10.1155/2022/3029582

**Published:** 2022-05-20

**Authors:** Chonnikant Visuthranukul, Sira Sriswasdi, Surapun Tepaamorndech, Yutthana Joyjinda, Puthita Saengpanit, Tanisa Kwanbunbumpen, Ekkarit Panichsillaphakit, Jaraspong Uaariyapanichkul, Sirinuch Chomtho

**Affiliations:** ^1^Pediatric Nutrition Research Unit, Division of Nutrition, Department of Pediatrics, Faculty of Medicine, Chulalongkorn University, Bangkok 10330, Thailand; ^2^Research Affairs, Faculty of Medicine, Chulalongkorn University, Bangkok 10330, Thailand; ^3^Center of Excellence in Computational Molecular Biology, Faculty of Medicine, Chulalongkorn University, Bangkok 10330, Thailand; ^4^National Center for Genetic Engineering and Biotechnology (BIOTEC), Khlong Luang, Pathum Thani 10210, Thailand; ^5^Department of Microbiology, Faculty of Medicine, Chulalongkorn University, Bangkok 10330, Thailand; ^6^WHO-CC for Research and Training on Viral Zoonoses, Faculty of Medicine, Chulalongkorn University, Bangkok 10330, Thailand; ^7^Thai Red Cross Emerging Infectious Diseases Health Science Centre, Bangkok 10330, Thailand; ^8^Division of Nutrition, Department of Pediatrics, King Chulalongkorn Memorial Hospital, The Thai Red Cross Society, Bangkok 10330, Thailand

## Abstract

**Background:**

Dysbiosis of intestinal microbiota may be linked to pathogenesis of obesity and metabolic disorders.

**Objective:**

This study compared the gut microbiome of obese Thai children with that of healthy controls and examined their relationships with host lifestyle, adiposity, and metabolic profiles.

**Methods:**

This cross-sectional study enrolled obese children aged 7–15. Body composition was evaluated using bioelectrical impedance analysis. Stool samples were analyzed by 16S rRNA sequencing using the Illumina MiSeq platform. Relative abundance and alpha- and beta-diversity were compared with normal-weight Thai children from a previous publication using Wilcoxon rank-sum test and ANOSIM. Relationships of gut microbiota with lifestyle activity, body composition, and metabolic profiles were assessed by canonical correlation analysis (CCA) and Spearman correlation.

**Results:**

The study enrolled 164 obese children with a male percentage of 59%. Mean age was 10.4 ± 2.2 years with a BMI *z*-score of 3.2 ± 1. The abundance of Bacteroidetes and Actinobacteria were found to be lower in obese children compared to nonobese children. Alpha-diversity indices showed no differences between groups, while beta-diversity revealed significant differences in the family and genus levels. CCA revealed significant correlations of the relative abundance of gut microbial phyla with sedentary lifestyle and certain metabolic markers. Univariate analysis revealed that Actinobacteria and *Bifidobacterium* were positively correlated with HDL-C and negatively correlated with body weight and screen time. Additionally, Actinobacteria was also negatively associated with fasting insulin and HOMA-IR. *Lactobacillus* showed positive correlation with acanthosis nigricans and adiposity. Cooccurrence analysis revealed 90 significant bacterial copresence and mutual exclusion interactions among 43 genera in obese children, whereas only 2 significant cooccurrences were found in nonobese children.

**Conclusions:**

The composition and diversity of gut microbiota in obese Thai children were different from those of their normal-weight peers. Specific gut microbiota were associated with lifestyle, adiposity, and metabolic features in obese children. An interventional study is needed to support causality between specific gut microbiota and obesity.

## 1. Introduction

Obesity is a major health concern with a dramatic increase in both developed and developing countries. According to the World Health Organization (WHO), over 1.6 billion adults are overweight, and more than 400 million adults are obese [1]. The worldwide prevalence of obesity in children and adolescents is also rapidly rising. In Thailand, 7.6%, and 9.0% of children and adolescents aged 3 to 18 years are overweight and obese, respectively [2]. Obese children are predisposed to develop severe comorbidities including type 2 diabetes, dyslipidemia, metabolic syndrome, and cardiovascular diseases [3].

While it is generally accepted that the development of obesity is caused by gene-environment interactions, physiological and environmental predispositions underlying obesity, and associated metabolic disorders are still unclear. Recent studies have suggested that intestinal microbiota is possibly linked to host-microbe interactions regarding energy harvest and regulation and plays a crucial role in the pathogenesis of obesity [4, 5]. The fundamental role of gut microbiota in regulation and pathogenesis of metabolic disorders has attracted great research interest in recent years.

Differences in gut microbial communities are correlated with obesity, which can lead to a reduction in the diversity of gut microbiota [5, 6]. Previous studies showed that obesity was associated with a specific profile of gut microbiota, including an increased Firmicutes/Bacteroidetes ratio [7, 8]. Other studies revealed significant associations between an increase in some bacterial groups, such as *Lactobacillus* [9] and *Faecalibacterium prausnitzii*, and obesity [10]. In contrast, *Bifidobacterium* was associated with leanness [10, 11]. Previous reports also detected gut microbial shifts in obese children. Gut microbiota composition was significantly different between obese Korean children and their healthy counterparts [8]. It was also demonstrated that the gut microbiota composition of obese and normal-weight Chinese children differed [7, 12]. Moreover, the associations between some gut bacterial phylum and genus with clinical features were reported [7, 8, 12]. The extensive diversity of gut microbiomes in racial groups could be explained by various factors including diet, lifestyle, genetic background, and geological locations [7, 13]. To the best of our knowledge, there are no such data for obese Thai children.

In the present study, we aimed to determine the composition and diversity of gut microbiota in obese Thai children compared to those of nonobese children. A secondary aim was to identify associations of composition and diversity of the microbiota with children's body composition, metabolic profiles, and lifestyle activity.

## 2. Methods

### 2.1. Subjects

This investigation was a cross-sectional study conducted from August 2017 to July 2020. The inclusion criteria were (1) children aged 7 to 15 years and (2) BMI > median + 2 SD based upon WHO growth reference [14]. The exclusion criteria were syndromic obesity and endocrine causes of obesity (e.g., hypothyroidism and growth hormone deficiency). Children were recruited for an obesity intervention trial at the King Chulalongkorn Memorial Hospital, Bangkok, Thailand. The study was approved by the Institutional Review Board for Human Subjects in the Faculty of Medicine, Chulalongkorn University (IRB 240/60). Written informed consent was obtained from all participants and their legal guardians. We compared the gut microbiota composition and diversity in 164 obese Thai children from our study to previously published data obtained from 45 nonobese Thai children [15]. The mean age of our cohort was 10.4 ± 2.2 years, and 59% of the subjects were male. The mean age, weight, height, and BMI for the nonobese children who lived in urban area (Bangkok, *n* = 17) and in rural area (Buriram, *n* = 28) were 10.5 ± 0.7 years, 45.6 ± 12.2 kg, 145.4 ± 11.1 cm, and 21.3 ± 4.0 kg/m^2^, respectively, and 9.8 ± 0.6 years, 34.4 ± 8.0 kg, 139.5 ± 7.4 cm, and 17.4 ± 2.9 kg/m^2^, respectively. Males were 81% and 43% of nonobese children in Bangkok and Buriram, respectively. Microbiome analysis in the previous study was completed on the variable region, V1–V2, of 16S rRNA sequencing using Illumina MiSeq v3 technology (Illumina Inc., San Diego, CA, USA).

### 2.2. Assessment of Dietary Intake, Physical Activity, and Exercise

Dietary intake was evaluated by a dietician using 24-hour dietary recall. The daily energy, percentage of energy distributions, fiber intake, and nutrients intake were calculated using INMUCAL-Nutrients Version 3 (the Institute of Nutrition, Mahidol University Calculation software) [16]. Physical activity and sedentary lifestyle were assessed by questionnaires and interviews by a research assistant.

### 2.3. Anthropometry and Body Composition

Anthropometric measurements, including body weight, height, waist, and hip circumferences, were collected by trained personnel. BMI was calculated as weight in kilograms divided by height squared in meters (kg/m^2^), and BMI z-scores were calculated based on WHO 2007 growth reference using the WHO AnthroPlus program [17]. Body composition was measured by bioelectrical impedance analysis (BIA) using the InBody 770 (InBody Co., Ltd., Chungcheongnam-do, Korea). Fat mass index (FMI) and fat-free mass index (FFMI) were calculated in the same manner as BMI [18].

### 2.4. Metabolic Profiles

Venous blood was obtained after a 12-hour fast to evaluate biochemical parameters. Fasting plasma glucose (FPG) was measured by the hexokinase method (Glucose, Architect; Abbott Laboratories, Irving, TX). Fasting insulin (FI) was measured using Immulite/Immulite 1000 Insulin assay (Immulite Analyzer; Abbott Laboratories). Serum total cholesterol, HDL-cholesterol (HDL-C), and triglycerides were measured by enzymatic colorimetric assay (Cholesterol, Architect; Ultra HDL, Architect; and Triglyceride, Architect; Abbott Laboratories). LDL-cholesterol (LDL-C) was measured by homogeneous liquid selective detergent (Direct LDL, Architect; Abbott Laboratories). Serum alanine aminotransferase (ALT) was determined according to the standard of International Federation of Clinical Chemistry (Alanine Aminotransferase, Architect; Abbott Laboratories). Homeostatic model assessment for insulin resistance (HOMA-IR) was calculated from (FI × FPG)/22.5, where FI and FPG were fasting insulin concentration (mU/l) and fasting plasma glucose (mmol/l), respectively [19].

### 2.5. Fecal Collection, DNA Isolation, and 16S PCR Amplification

Participants were instructed to collect fecal samples in their households. Sterile stool collection kits, containers, and temperature-controlled packages were provided. A fresh stool was placed into a half of 50 ml sterile container and double-sealed in a zip-lock bag. The sample was stored in the freezer compartment of a home refrigerator, delivered to the laboratory within 24 hours, and stored at −80°C until use. Fecal samples were resuspended in InhibitEX Buffer (QIAGEN, Germany), incubated at 70°C for 5 min, and centrifuged at 20,000 × *g* for 1 min. Supernatant was collected for DNA isolation using QIAamp fast DNA Stool Mini Kit (QIAGEN, Germany) according to the manufacturer's instructions. DNA integrity and concentration were quantitated using the Qubit dsDNA HS assay (Life Technologies, USA) on a Qubit 4 Fluorometer (Life Technologies, USA). The V3–V4 hypervariable region of the 16S ribosomal RNA (rRNA) gene was PCR-amplified using specific primers [20]. The PCR products were subjected to gel purification using the NucleoSpin Gel and PCR Clean-Up Kit (Macherey-Nagel, Germany) according to the manufacturer's instructions.

### 2.6. 16S Sequencing and Data Analysis

The DNA amplicon of the bacterial 16s rRNA gene was PCR-amplified using a specific primer targeting the V3–V4 region. The PCR products were run on agarose gel electrophoresis followed by gel extraction, and purification using the NucleoSpin Gel and PCR Clean-Up Kit (Macherey-Nagel). The Illumina's indices were added to both ends of the PCR products to allow for multiplexing samples. Indexed PCR products were then cleaned using Agencourt AMPure XP beads (Beckman Coulter Inc.). DNA concentration was quantitated using a Qubit dsDNA HS Assay Kit (Life Technologies). The exact size of indexed libraries was checked with QIAxcel capillary electrophoresis (QIAGEN, Germany) before library pooling, and sequencing using paired-ends (2 × 301 bp) on Illumina MiSeq with Illumina V3 Reagent Kit.

### 2.7. Sequencing Data and Taxonomic Analysis

Readings from Illumina paired-end sequencing were trimmed, merged, filtered, and subsequently assigned to taxonomic classes using the QIIME2 platform release 2020.8 [21]. Initial quality screening was performed using FastQC [22] to identify the number of base positions with low confidence. The first 10 positions of all reads, the last 30 positions of forward reads, and the last 70 positions of reverse reads were trimmed. Reads with more than two expected errors in either the forward or reverse strand and reads that could be mapped to multiple origins (chimeric reads) were removed using the DADA2 [23] module in QIIME2. On average, 30% of the 267,000 reads in each sample passed the quality filtering step and 24.75% passed the chimera screening. We found that trimming more or fewer read positions both led to significantly less number of reads that passed the whole quality filtering pipeline. While trimming reads more aggressively yielded reads with higher quality (40% passed the quality filtering step), many reads became so short that the forward and reverse fragments did not overlap (only 10% passed the merging step). Conversely, trimming fewer base positions generated reads with unacceptable quality (only 15% passed the quality filtering step). The DADA2 module also clustered reads into 7,211 representative sequences.

### 2.8. Taxonomic Class Assignment

Representative sequences that passed the quality filtering were assigned to taxonomic classes using the *silva-132-99-nb* pretrained Naïve Bayes model available on QIIME2. This classifier was trained using full-length 16S rRNA sequences from SILVA database [24]. The confidence threshold was set at 0.7 as default. We also built a custom taxonomic classifier using only the V3–V4 hypervariable regions of 16S rRNA to assign sequences to taxa but did not observe any noticeable differences from the results obtained using the prebuilt model. Overall, more than 98% of the 7,211 representative sequences could be mapped at phylum, class, order, and family levels, while 87% could be mapped to a genus and 35% could be mapped to a species. Our subsequent statistical analyses focused on the phylum and genus levels.

### 2.9. Taxonomic Composition and Diversity Analysis

Analysis of composition of microbiomes (ANCOM) [25] was used to test whether taxonomic compositions at the phylum, family, and genus levels differed significantly between obese and nonobese Thai children [15]. ANCOM evaluates multiple per-taxon Wilcoxon rank-sum tests simultaneously to address the fact that changes in abundance of one taxon would affect the abundance of others because taxonomic composition sums to one. Analysis of similarities (ANOSIM) [26] was used to test whether beta-diversities between obese and nonobese children significantly differed as measured by Bray–Curtis dissimilarity. To identify the most appropriate dataset of nonobese Thai children to be compared with our obese cohort using the described ANCOM and ANOSIM methods, several past gut microbiome studies of Thai children were examined, and the Kisuse et al. study [15] was selected because it showed no significant difference in phyla profiles from our dataset.

### 2.10. Diversity Calculations

The sample-taxa frequency table was re-summarized at the phylum and genus levels by summing read counts belonging to the same phylum or genus together. Operational taxonomic units (OTUs) with unassigned phylum or genus were discarded from further analyses at the respective levels (i.e., OTUs with unassigned genus but assigned phylum were included in the analyses at the phylum level). Shannon, Simpson, and Chao1 alpha-diversity indices were calculated for each sample based on their mathematical definitions [27]. To evaluate beta-diversity structure, a phylogenetic tree containing all identified taxa was reconstructed using the *phylogeny* module in QIIME2. UniFrac distances between samples were calculated using the *diversity-lib* module in QIIME2.

### 2.11. Cooccurrence Network Reconstruction

For cooccurrence analysis, rare genera with average abundance of less than 0.1% were excluded because they are typically identified with very few reads, and their abundances may not be sufficiently estimated by our sequencing data. This filter reduced the number of genera from 296 to 72, respectively. Cooccurrence of two genera across 164 patient samples was calculated using Jaccard index which is the ratio of the number of samples that contain both genera to the number of samples that contain at least one of the genera. A hypergeometric model [28] was then used to test whether cooccurrence of each pair of phyla or genera was significant. A Bonferroni adjusted *P* value cutoff at 0.05 was applied.

### 2.12. Statistical Analysis

Descriptive data analysis was performed using SPSS Statistics version 23 (SPSS Inc., Chicago, IL, USA). The normality of data was tested using the Shapiro–Wilk test. Normally distributed data were presented as means with SD, while non-normally distributed data were reported as medians with quartiles (Q1, Q3). A *P* value of less than 0.05 was considered as statistically significant. Spearman rank correlations were calculated between the abundances of each taxon and the values of each clinical variable across 164 samples. *P* values for each correlation value were estimated using a permutation test (5,000 shuffle) against the null hypothesis in which there was no association between the two variables. The overall false discovery rate was controlled within 5% by Benjamini–Hochberg procedure. Partial Spearman correlations were calculated for comparisons between taxa and metabolic profiles adjusted for effects from age, sex, and BMI using the *partial_corr* function in the pingouin Python library. Canonical correlation analyses (CCA) between microbial abundances and clinical variable groups were performed using *CCA* function in scikit-learn Python library [29]. Significance of each CCA component was evaluated using Wilks' lambda [30] which tests the null hypothesis that the *n*-th canonical correlation and all subsequent correlations are zero. CCA were performed on the clinical and metabolic variable groups.

## 3. Results

### 3.1. Participants' Demographic Data, Dietary Intake, Physical Activity, and Metabolic Profiles

A total of 164 obese children were enrolled in this study. The demographic data are shown in Table 1. Mean age and BMI z-score were 10.4 ± 2.2 years and 3.2 ± 1.0, respectively. Males represented 59% of participants. Acanthosis nigricans was detected in 80% of children, and 63.6% were at Tanner stage 1. Mean fat mass index (FMI), fat-free mass index (FFMI), and body fat percentage were 11.8 ± 2.9, 16.1 ± 2.1 kg/m^2^, and 41.7 ± 5.1%, respectively. Mean energy intake was 1,450 ± 537.9 kcal/day. Dietary fiber intake was about 2.8 g/1000 kcal. Caloric distribution was 48% carbohydrate, 16% protein, and 36% fat. The participants were classified into participating in high (49%), moderate (40%), and low (8%) intensity exercise regimens. Only 3% of participants did not report doing any exercise. The questionnaire on lifestyle patterns revealed that the median time spent for screen time, such as watching television or using electronic devices, was 7 (Q1 2, Q3 8) hours/day during weekdays, and 8 (4, 10) hours/day during weekends. Median sedentary activity was 4 (2, 7) hours/day. The prevalence of high SBP, hypercholesterolemia, high LDL-C, low HDL-C, and hypertriglyceridemia was 9%, 37%, 42%, 10%, and 11%, respectively.

### 3.2. Gut Microbiota Composition and Diversity in Obese Thai Children

**Figure 1:**
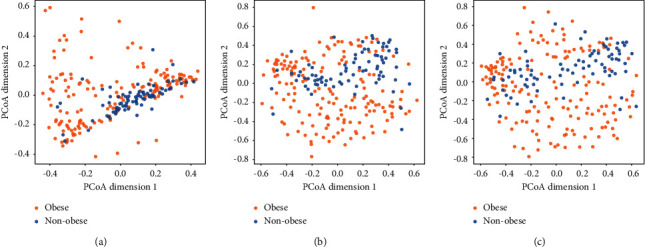
Beta-diversity of bacterial phylum (a), family (b), and genus (c) in obese and nonobese children. PCoA based on Bray–Curtis dissimilarity was performed with the observation showing no significant difference at the phylum level; however, there were significant differences in gut microbial community structure at the family and genus levels between obese and nonobese children (*P*=0.012 and *P*=0.003). It should be noted that parts of these observations could be due to differences in sample processing and choice of hypervariable region between our study and previous works. Obese children are shown in orange; nonobese children are shown in blue. PCoA: principal coordinate analysis.

Stool samples were collected from 164 participants and underwent 16S rRNA sequencing and analyses. The alpha-diversity indices (Shannon, Simpson, and Chao1 index) showed no significant differences between obese and nonobese children. For beta-diversity evaluation, PCoA based on Bray–Curtis dissimilarity tests was performed and showed no significant differences at the phylum levels between obese and nonobese children (Figure 1(a)). There were significant differences in gut microbial community structures at the family and genus levels between obese and nonobese children (*P*=0.012 and *P*=0.003) (Figures 1(b) and 1(c)). It should be noted that some of these observational differences could be due to differences in sample processing and choice of hypervariable regions between our study and the comparison study.

**Figure 2:**
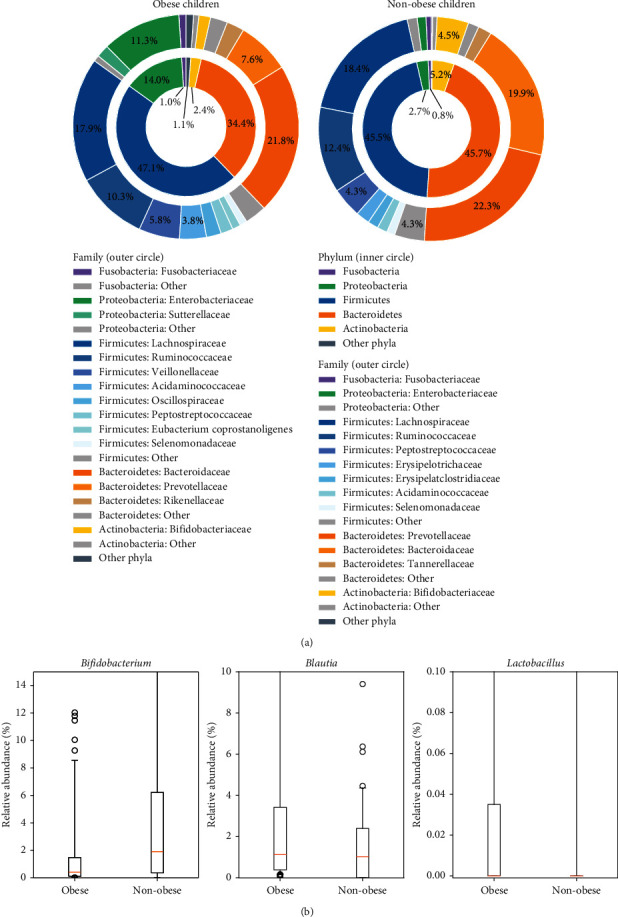
The relative abundance of bacterial compositions in obese and nonobese children at the phylum and family levels is shown in (a). The inner circle demonstrates the composition at the phylum level, and the outer circle demonstrates the composition at the family level. In the obese children, the most abundant bacterial population at the phylum level was Firmicutes (47.1%). In the nonobese children, the dominant gut bacteria contained Firmicutes and Bacteroidetes of about 46.0%. The obese children contained significantly lower numbers of Bacteroidetes and Actinobacteria than nonobese controls (*P* < 0.001, Wilcoxon rank-sum test). Obese children showed a greater average proportion of Proteobacteria and Fusobacteria than nonobese children (*P* < 0.001). No significant difference in Firmicutes was observed between obese and nonobese children. The compositional differences in obese and nonobese children at the genus level are shown in (b). Orange lines indicate the median, and black boxes indicate the 1^st^–3^rd^ interquartile range. Whiskers extend beyond the interquartile range by 1.5 times, starting from Q1 − 1.5*∗*(Q3 − Q1) to Q3+1.5*∗*(Q3 − Q1). Black circles indicate individual outlying data points. The relative abundance of *Bifidobacterium* was significantly higher in the nonobese children than in the obese children (*P* < 0.0001), but *Blautia* and *Lactobacillus* in the nonobese children were significantly lower than those in the obese children (*P*=0.0035 and *P*=0.0053).

The relative abundance of the bacterial composition by the phylum and family levels is illustrated in Figure 2(a). In the obese children, the most abundant bacterial population at the phylum level was Firmicutes with an average percent richness of 47.1%. The average numbers of Bacteroidetes, Proteobacteria, Actinobacteria, and Fusobacteria were 34.4%, 14.0%, 2.4%, and 1.0%, respectively. In comparison, in nonobese children, the dominant gut bacteria were almost equivalent between Firmicutes and Bacteroidetes at 46.0% of the total bacterial population. Actinobacteria, Proteobacteria, and Fusobacteria were also detected in nonobese children at relative abundances of 5.2%, 2.7%, and 0.8%, respectively. The obese children had a significantly lower relative number of Bacteroidetes and Actinobacteria than the nonobese control (*P* < 0.001, Wilcoxon rank-sum test). In contrast, obese children showed significantly greater abundance of the average proportion of Proteobacteria and Fusobacteria than the nonobese children at the significant level (*P* < 0.001). No significant difference in Firmicutes was observed between populations. The ratio of Firmicutes to Bacteroidetes (F/B) was not significantly different in obese children compared to nonobese children. However, the ANCOM test demonstrated that there were significant differences between obese and nonobese children in all bacterial phyla (*P* < 0.0001).

Among genera-level taxa, the relative abundance of *Bifidobacterium* was significantly higher in the nonobese compared to the obese children (*P* < 0.0001), while the abundance of *Blautia* and *Lactobacillus* was significantly lower in the nonobese children (*P*=0.0035 and *P*=0.0053) (Figure 2(b)). The relative abundance of *Collinsella, Lachnospiraceae NK4A136* group, and *Clostridioides* in the nonobese children was not significantly higher than in the obese children.

### 3.3. Associations of Gut Microbiota with Dietary Intake, Lifestyle Activity, Body Composition, and Metabolic Profiles

The association of gut microbiota composition in major phyla with dietary intake, lifestyle activity, body composition, and metabolic profiles was determined using CCA function. The results showed a significant correlation between the relative abundance of gut microbial phyla and sedentary lifestyle (*r* = 0.64, *P* < 0.0001) (Supplementary Figure S1(a)). The gut microbial phyla were also significantly associated with certain metabolic markers, including FPG, TG, HDL-C, LDL-C, and ALT (*r* = 0.31, *P*=0.023) (Supplementary Figure S1(b)). However, no significant relationship between bacterial composition at the phylum levels with dietary intake, BMI *z*-score, and adiposity was observed.

Any significant correlation of gut microbial phyla, family, and genus with patient-level variables was examined by the univariate analysis. At the phyla level, Actinobacteria was negatively associated with body weight and weekday screen time (*r* = −0.16, *P*=0.04; *r* = −0.46, *P* < 0.0001). No correlations between any bacterial phyla and adiposity were observed. After adjusting for the confounding effects from age, sex, and BMI, Actinobacteria showed the strongest correlations with metabolic profiles including HDL-C (*r* = 0.15, *P*=0.025), FI (*r* = −0.14, *P*=0.044), and HOMA-IR (*r* = −0.14, *P*=0.042). Firmicutes was not correlated with BMI z-score or any metabolic profiles.

**Figure 3:**
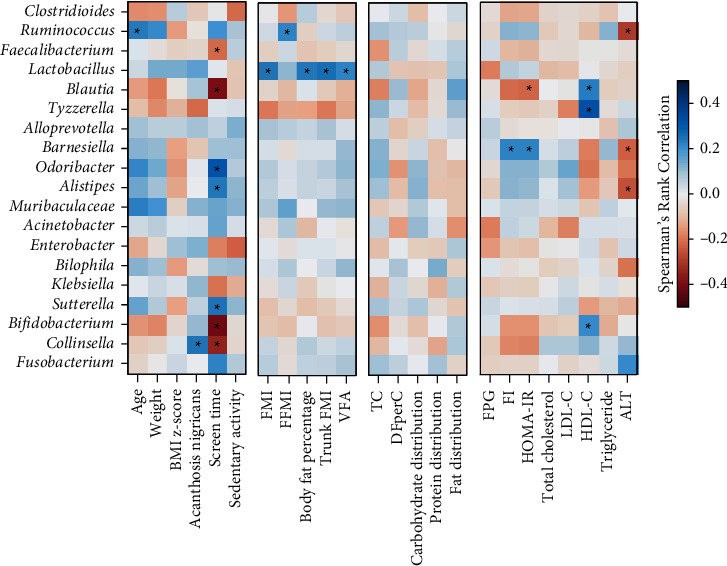
The relationships of the bacterial taxa at the genus level and clinical data, lifestyle activity, dietary intake, and metabolic profiles were determined using Spearman correlation and visualized using a heatmap. The color of the heatmap shows positive (red) and negative correlations (blue). Significant associations are marked with asterisks. ALT: alanine aminotransferase; DFperC: dietary fiber intake, g per 1,000 kcal; FFMI: fat-free mass index; FMI; fat mass index; FI: fasting insulin; FPG: fasting plasma glucose; HDL-C: high density lipoprotein cholesterol; HOMA-IR: Homeostatic Model Assessment for Insulin Resistance; LDL-C: low density lipoprotein cholesterol; TC: total calories; VFA: visceral fat area.

At the genus level, *Bifidobacterium* was positively correlated with HDL-C (*r* = 0.18, *P*=0.013 after accounting for confounders) but negatively associated with body weight (*r* = −0.16, *P*=0.017), total energy intake (*r* = −0.15, *P*=0.021), and screen time (*r* = −0.40, *P*=0.0002) (Figure 3). *Blautia* was positively associated with the proportion of fat in the diet (*r* = 0.16, *P*=0.020). *Lactobacillus* and *Collinsella* showed significant positive correlations with acanthosis nigricans (*r* = 0.17, *P*=0.019; *r* = 0.24, *P*=0.001). The relationship of gut microbiota at the genus level with body composition indices showed that *Lactobacillus* was significantly and positively associated with fat mass (FM) (*r* = 0.20, *P*=0.009), fat mass index (FMI) (*r* = 0.24, *P*=0.0002), body fat percentage (*r* = 0.24, *P*=0.0004), trunk FMI (*r* = 0.25, *P*=0.0002), and visceral fat area (VFA) (*r* = 0.22, *P*=0.002).

### 3.4. Cooccurrence Analysis

**Figure 4:**
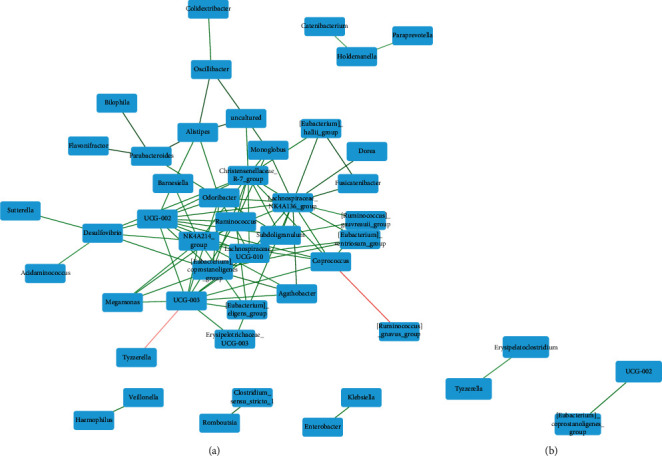
Cooccurrence analysis was performed to determine the interactions between gut microbiota at the genus level in obese (a) and nonobese (b) children. The color of the edges shows either positive (green) or negative (red) associations between genera. The color intensity of the edges indicates the relative strength of the association. Cooccurrences with a *P* value >0.05 were removed. This analysis revealed 90 statistically significant copresence (positive) and mutual exclusion (negative) bacterial interactions among 43 genera in obese children. In contrast, the cooccurrence network at the genus level for nonobese children found only two statistically significant bacterial cooccurrences.

To explore the interactions between gut microbiota in obese and nonobese children, a cooccurrence analysis was performed at the genus level. This analysis revealed 90 statistically significant bacterial copresence (positive) and mutual exclusion (negative) interactions among 43 genera in obese children (Figure 4(a) and Table 2). An abundance of genera belonging to Bacteroidetes and Firmicutes showed higher positive intra-phylum cooccurrence in the obese children (*P* < 0.001, Wilcoxon rank-sum test). The *Lachnospiraceae NK4A136* group showed copresence with many other bacteria in obese children. In contrast, the cooccurrence network at the genus level for nonobese children showed only two statistically significant bacterial cooccurrences of *Ruminococcaceae UCG-002* and *Eubacterium coprostanoligenes* group, and of *Erysipelatoclostridium* and *Tyzzerella* (Figure 4(b)). Compared with 10,000 random networks, bacterial cooccurrence in the obese children was significantly clustered (*P* < 0.0001, cluster coefficient = 0.46).

## 4. Discussion

This present study determined the composition and diversity of gut microbiota in obese Thai children, compared to nonobese Thai children, and examined microbial associations with clinical and metabolic parameters.

### 4.1. Bacterial Community and Diversity

This study examined 164 stool samples from obese children and adolescents. The results showed that gut microbial alpha-diversity, an index reflecting the variety of microbial species, from gastrointestinal tracts of obese children showed no difference compared to nonobese children. This finding was consistent with previous studies [7, 31]. However, the beta-diversity analysis used to assess the heterogeneity of gut microbiota between samples in each group found significant disparity. Our PCoA based on a Bray–Curtis dissimilarity matrix revealed a shifted gut microbiota profile between obese and nonobese children at the family and genus levels, as in a previous study [12]. This finding indicates that gut microbial composition might be associated with the pathogenesis of obesity. The subtle difference in beta-diversity that was detected between both populations at the phylum level is in contrast to the significant changes in microbial abundance suggested by Wilcoxon rank-sum test and ANCOM test. As a result, we further analyzed the relationships of all the phenotypic data and gut microbiota at the phylum level. We found sedentary lifestyle and metabolic profiles associated with certain gut microbiota at the phylum level in obese population. These observations were supported by significant differences in beta-diversity between the obese and nonobese children at the family and genus levels.

The results of the gut microbiota composition analysis showed that the predominant phylum was Firmicutes followed by Bacteroidetes, Proteobacteria, Actinobacteria, and Fusobacteria in obese children, similar to studies in Italy and Japan [32, 33]. Studies of obese Chinese children and adolescents showed that both Firmicutes and Bacteroidetes were the two major bacterial taxa with the same relative abundance [7, 12]. Studies in western countries have observed a similar pattern [31].

Our study revealed a difference in gut bacterial composition in obese children compared to nonobese children, who had both Firmicutes and Bacteroidetes as the predominant taxa. A difference in Firmicutes abundance between obese and nonobese children was not found in our study, as in previous studies [12, 34]. Although the F/B ratio in the present study had a tendency to be higher in obese children, it was not significantly different between obese and nonobese children, similar to other studies [12, 35]. Other researchers have previously demonstrated that the F/B ratio significantly increases in obese children [36, 37]. These inconsistent findings may be explained by the impact of gut microbiota on obesity being far more complicated than the imbalance or interaction of only two phyla. We propose that the F/B ratio might not be a good marker of gut microbiota in pediatric obesity. Moreover, we found that obese children had a lower abundance of Actinobacteria but had a higher abundance of Proteobacteria compared to nonobese children. Several microbial genera belonging to these two phyla have crucial functions in the body, but these differences were not reported in relevant previous studies in obese children [8, 12]. In contrast, a study from Mexico showed that obese children and adolescents had higher relative abundance of both Actinobacteria and Proteobacteria [31]. These inconsistent results may be due to differences in genetic, environmental, and dietary factors. At the genus level, *Bifidobacterium* was present in significantly lower abundance in obese children, as found in a previous study [12]. However, *Lactobacillus* and *Blautia* showed higher abundance in obese children, as in the study from Mexico [31, 38].

### 4.2. The Relationship of Gut Microbiota with Lifestyle and Metabolic Profiles in Obese Children

The relationship of gut microbiota with lifestyle and metabolic profiles was observed in this study. Healthy dietary habits and physical activity have been shown to impact obesity and metabolic outcomes [39]. Recent studies have also demonstrated the beneficial effects of exercise on reshaping microbiota composition in animal and adult models [40] suggesting that increased exercise and decreased sedentary activity in obese patients would be a potential strategy to modulate intestinal bacterial composition and improve metabolic status.

CCA results found significant correlations of certain gut microbial phyla with sedentary activity and metabolic profiles. These findings could give crucial insight into the host-microbe relationship. Therefore, we further explored the association between specific gut microbiota and those variables by univariate analysis. A positive correlation of Actinobacteria and HOMA-IR was consistent with a previous study [8]. This present study is the first to report on a relationship between Actinobacteria and sedentary lifestyle. Previous studies in obese children have reported a correlation of Firmicutes and Bacteroidetes with obesity and certain metabolic profiles, but no association with lifestyle activity has been found.

The associations at the genus level showed that *Bifidobacterium* was positively correlated with HDL-C, which was compatible with Actinobacteria as it belongs to this phylum. However, *Bifidobacterium* was negatively correlated with body weight, total energy intake, and screen time. These results were consistent with a previous study showing that *Bifidobacterium* decreased in obese individuals compared to normal-weight people [41]. The researchers demonstrated that a high-fat diet leads to a decrease in the number of *Bifidobacterium* spp. and caused inflammation, obesity, insulin resistance, while the number of *Bacteroides* and *Clostridium* spp. increased with such a diet [42, 43]. The present study found a negative correlation between *Bifidobacterium* and total energy intake that might indirectly result from high-fat intake by obese children.

Regarding lifestyle activity, the use of electronic devices is becoming a major driver in the reduction of physical activity in the context of pediatric obesity [3]. The present study found that the long-time use of electronic devices in obese children averaged 6–8 hours a day. Increased use of electronic devices was associated with a decrease in *Bifidobacterium.* One study reported that electronic device usage close to bedtime may disrupt sleep patterns, a factor associated with obesity [44]. This underscores that all factors including diet, lifestyle, and physical activity, not just microbiota, should be taken into account in understanding obesity [45].

We found that *Lactobacillus* was correlated with acanthosis nigricans and adiposity including FM, FMI, body fat percentage, trunk FMI, and VFA with an increased relative abundance of this genus in obese children. These results were consistent with previous studies finding that *Lactobacillus* was associated with weight gain in humans [9, 46, 47] and animals [46]. Additionally, previous studies using probiotics such as *Lactobacillus* spp. reported weight gain with the use of *L. rhamnosus*. Some studies have shown an association between *L. reuteri* and obesity [48, 49]. One possible reason for this weight gain is that *L. reuteri* might improve the ability to absorb and process nutrients in the gut [50]. In contrast, some *Lactobacillus* species were associated with weight loss both in obese humans and in obese animals [51, 52], indicating that different *Lactobacillus* species produce different effects on weight that may be host-specific. Further studies are needed to clarify the role of *Lactobacillus* in human energy harvest and weight regulation.

Bacteria of the genus *Collinsella* were associated with acanthosis nigricans in obese children in our study. This genus was demonstrated to be positively correlated with insulin [53] and elevated in type 2 diabetes patients [54]. High levels of *Collinsella* might imply the possibility of insulin resistance in obese children which would support our findings. *Blautia* was associated with fat distribution of total energy intake in obese children. A previous study found that this genus was related to a high-fat diet causing obesity in mice [55].

### 4.3. Cooccurrence of Gut Microbiota

Cooccurrence analysis of gut microbiota revealed a significant difference between obese and nonobese children. We found that obese children had denser clustering of interactions between gut microbial genera that were similarly observed in previous studies [8, 31, 32]. *Lachnospiraceae NK4A136* group, a member of family Lachnospiraceae and phylum Firmicutes, showed the highest number of positive interactions, which suggested that this genus member has mutualistic relationships with other bacteria. The previous report showed that the members of the Lachnospiraceae family were correlated with type 2 diabetes and obesity [56, 57]. Moreover, a study showed that Lachnospiraceae-co-abundance groups were associated with increased risk of obesity [58]. Lachnospiraceae is known to play a role in SCFA production [59]. Although exogenous SCFA introduction could attenuate body fat deposition while promoting fat oxidation, SCFAs derived from gut microbiota could provide additional energy for the host [60]. A previous study reported that fecal transplant from obese to germ-free mice increased SCFA contents associated with their body weight gain [11]. In lipid metabolism, SCFAs could be the substrate of long-chain fatty acids in triglyceride synthesis. Accordingly, it was not surprising that our study demonstrated the presence of Lachnospiraceae and others in the obese group compared to the nonobese group. In fact, Lachnospiraceae could be enriched by high-fat diets. An increase of this bacterial family is likely to promote complex carbohydrate breakdown into SCFAs [59]. In obese children, Lachnospiraceae may be enriched by the host's diet, especially high-fat contents, leading to increased efficiency in SCFA production, and more energy extraction to the host. Two negative interactions indicating competition between microbial taxa were also identified: one between *Prevotellaceae UCG-003* and *Tyzzerella* and one between *Coprococcus* and *Ruminococcus gnavus*. Similar to previous reports [8, 31, 32], nonobese children showed only two cooccurrences and no mutual exclusion interactions. This may be explained by the fact that there is no common condition among the nonobese children that would make specific microbial cooccurrence remarkable. Moreover, large and dense networks may help a particular group of microbiota gain ecological dominance for more space and regulate host-microbe interactions inside the gut of obese individuals.

### 4.4. Strengths and Limitations of the Study

This study is the largest investigation documenting the gut microbiota composition in obese children. Since it is limited to only Thai children, the study is less influenced by ethnicity and some environmental factors. In addition to comparing the microbiota composition and diversity between obese and nonobese children, the relationships of microbial composition to clinical data, dietary intake, lifestyle activity, body composition, and metabolic profiles analyzed through both CCA and univariate analysis were determined. We also presented the results of a cooccurrence analysis of the gut microbiota in obese and nonobese children that can lead to an increased understanding of mutualistic relationships between bacterial genera. The limitations of this study include a small sample of nonobese children from the other study with some limitations due to the difference in hypervariable regions analyzed. Some children lived in the same area, but some did not. In addition, since the study employed a cross-sectional study design, support for any notion of causality is limited. Therefore, an interventional study to normalize dysbiosis of gut microbiota as a therapeutic target of pediatric obesity and its complications is warranted.

## 5. Conclusions

The composition of gut microbiota in obese Thai children differed from that in nonobese children. The beta-diversity also showed significant differences in gut microbial community structure between obese and nonobese children. Our study found significant mutualistic relationships between bacterial genera in the gut microbiomes of obese children that are completely absent in nonobese children. In obese children, Actinobacteria had a negative association with clinical features linked to obesity, sedentary lifestyle, and metabolic profiles related to insulin resistance, which was compatible with the specific genus *Bifidobacterium*. *Lactobacillus* also showed a positive relationship with insulin resistance, acanthosis nigricans, and adiposity. Restoring gut microbial dysbiosis could be part of a therapeutic strategy to target pediatric obesity. Any interventions to modulate beneficial microbes and their metabolites could be advantageous.

## Figures and Tables

**Table 1:** Demographic data of obese participants (*n* = 164).

Parameters	
Age, years	10.4 ± 2.2
Male gender (%)	59
Total nutrient intake	
Energy intake (kcal/day)	1,450 ± 537.9
Protein intake (g/kg/day)	1.6 ± 0.6
Dietary fiber (g/1,000 kcal)	2.8 ± 2
Fat intake (g/day)	56.1 ± 25
Energy distribution (%C : P : F)	48 : 16 : 36
Exercise	
Low intensity (min/wk)^1^	75 (0, 150)
Moderate intensity (min/wk)^2^	60 (5.5, 150)
Sedentary activity (hr/day)	4 (2, 7)
BMI for age z-score	3.2 ± 1
Waist circumference (cm)	89.7 ± 10.7
SBP (mmHg)	116 ± 10
Acanthosis nigricans (%)	80
Body composition (BIA)	
FMI (kg/m^2^)	11.8 ± 2.9
FFMI (kg/m^2^)	16.1 ± 2.1
Body fat percentage (%)	41.7 ± 5.5
Trunk FMI (kg/m^2^)	5.7 ± 1.4
VFA (cm^2^)	129.4 ± 40.3
Metabolic profiles	
Total cholesterol (mg/dL)	189.6 ± 31.7
LDL-C (mg/dL)	128.9 ± 31.6
HDL-C (mg/dL)	51.2 ± 9.6
Triglyceride (mg/dL)	101.6 ± 41.8
ALT (U/L)	30.4 ± 24.9
FPG (mg/dL)	82.6 ± 5.9
FI (mU/L)	14.5 ± 13.4
HOMA-IR	3.0 ± 2.7

Data shows means ± SD, median (Q1, Q3), or %. ^1^Low intensity (min/wk) was walking from home to school or walking from one place to another for at least 10 minutes. ^2^Moderate intensity (min/wk) was brisk walking or riding a bicycle continuously for at least 10 minutes. Sedentary activity was defined as a type of lifestyle involving little or no physical activity. ALT: alanine aminotransferase; BIA: bioelectrical impedance analysis; C: cholesterol; FPG: fasting plasma glucose; FI: fasting insulin; FMI: fat mass index = fat mass (kg)/height (m^2^); FFMI: fat-free mass index = fat-free mass (kg)/height (m^2^); HDL-C: high density lipoprotein cholesterol; HOMA-IR: Homeostatic Model Assessment for Insulin Resistance; LDL-C: low density lipoprotein cholesterol; SBP: systolic blood pressure; VFA: visceral fat area.

**Table 2:** List of bacterial genera for Figure 4.

Obese children (Figure 4(a))		Nonobese children (Figure 4(b))	
No.	Bacterial genus	No.	Bacterial genus
1	*[Eubacterium]_coprostanoligenes_*group	1	*[Eubacterium]_coprostanoligenes_*group
2	*[Eubacterium]_eligens_*group	2	*Erysipelatoclostridium*
3	*[Eubacterium]_hallii_*group	3	*Tyzzerella*
4	*[Eubacterium]_ventriosum_*group	4	*UCG-002*
5	*[Ruminococcus]_gauvreauii_*group		
6	*[Ruminococcus]_gnavus_*group		
7	*[Ruminococcus]_torques_*group		
8	*Acidaminococcus*		
9	*Acinetobacter*		
10	*Agathobacter*		
11	*Akkermansia*		
12	*Alistipes*		
13	*Alloprevotella*		
14	*Anaerostipes*		
15	*Bacteroides*		
16	*Barnesiella*		
17	*Bifidobacterium*		
18	*Bilophila*		
19	*Blautia*		
20	*Butyricicoccus*		
21	*CAG-352*		
22	*Catenibacterium*		
23	*Christensenellaceae_R-7_*group		
24	*Clostridioides*		
25	*Clostridium_sensu_stricto_1*		
26	*Colidextribacter*		
27	*Collinsella*		
28	*Coprococcus*		
29	*Desulfovibrio*		
30	*Dialister*		
31	*Dorea*		
32	*Enterobacter*		
33	*Erysipelotrichaceae_UCG-003*		
34	*Escherichia-Shigella*		
35	*Faecalibacterium*		
36	*Flavonifractor*		
37	*Fusicatenibacter*		
38	*Fusobacterium*		
39	*Haemophilus*		
40	*Holdemanella*		
41	*Incertae_Sedis*		
42	*Klebsiella*		
43	*Lachnoclostridium*		
44	*Lachnospira*		
45	*Lachnospiraceae_NK4A136_*group		
46	*Lachnospiraceae_UCG-010*		
47	*Lactobacillus*		
48	*Megamonas*		
49	*Megasphaera*		
50	*Monoglobus*		
51	*Muribaculaceae*		
52	*NK4A214_*group		
53	*Odoribacter*		
54	*Oscillibacter*		
55	*Parabacteroides*		
56	*Paraprevotella*		
57	*Parasutterella*		
58	*Peptostreptococcus*		
59	*Phascolarctobacterium*		
60	*Prevotella*		
61	*Romboutsia*		
62	*Roseburia*		
63	*Ruminococcus*		
64	*Streptococcus*		
65	*Subdoligranulum*		
66	*Sutterella*		
67	*Tyzzerella*		
68	*UBA1819*		
69	*UCG-002*		
70	*UCG-003*		
71	*Uncultured*		
72	*Veillonella*		

Our taxonomy annotation database assigned *Muribaculaceae* as the genus names to member of this family.

## Data Availability

Data described in the manuscript will be made available upon request pending application and approval from the corresponding author.

## Supplementary Materials

Supplementary Materials Supplementary Figure S1: the associations of gut microbiota composition in major phyla with sedentary activity (a) and major phyla with metabolic profiles (FPG, TG, HDL-C, LDL-C, and ALT) (b) were determined using CCA function. The results showed a significant correlation between relative abundance of gut microbial phyla and sedentary lifestyle (*r* = 0.64, *P* < 0.0001) as well as metabolic profiles (*r* = 0.31, *P*=0.023). ALT: alanine aminotransferase; CCA: canonical correspondence analysis; HDL-C: high density lipoprotein cholesterol; LDL-C: low density lipoprotein cholesterol.
