# Supplementary material for: Association of Human Intestinal Microbiota with Lifestyle Activity, Adiposity, and Metabolic Profiles in Thai Children with Obesity
Source: J Nutr Metab. 2022 May 20;2022:3029582. doi: 10.1155/2022/3029582 (PMC9146442; doi:10.1155/2022/3029582)

**Supplementary Figure S1** The associations of gut microbiota composition in major phyla with sedentary activity (a), and major phyla with metabolic profiles (FPG, TG, HDL-C, LDL-C, and ALT) (b) were determined using CCA function. The results showed a significant correlation between relative abundance of gut microbial phyla and sedentary lifestyle (r = 0.64, P < 0.0001) as well as metabolic profiles (r = 0.31, P = 0.023).

ALT, alanine aminotransferase; CCA, Canonical Correspondence Analysis; HDL-C, high density lipoprotein cholesterol; LDL-C, low density lipoprotein cholesterol


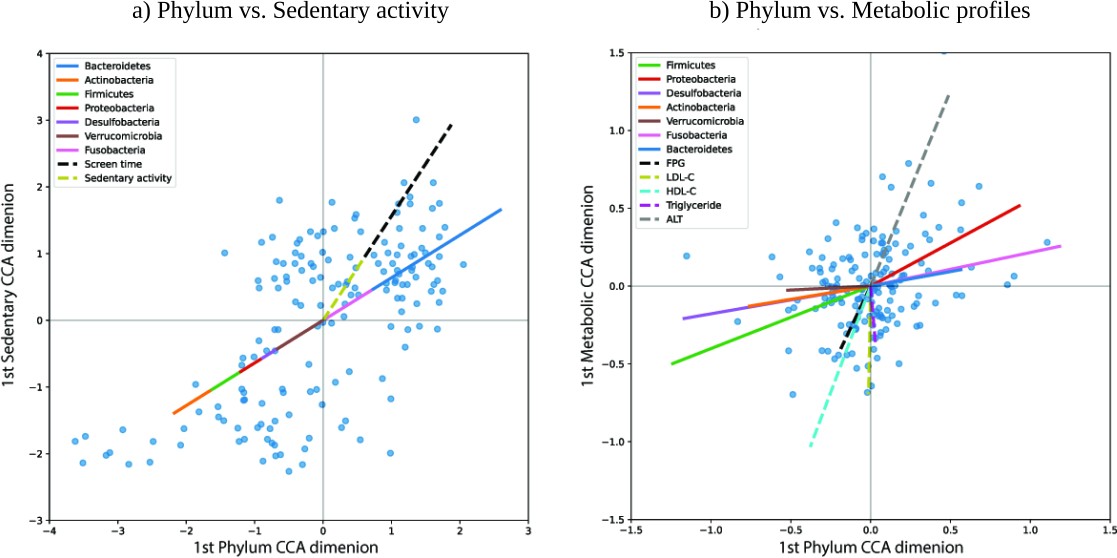

Supplement: Supplementary Materials — Supplementary Figure S1: the associations of gut microbiota composition in major phyla with sedentary activity (a) and major phyla with metabolic profiles (FPG, TG, HDL-C, LDL-C, and ALT) (b) were determined using CCA function. The results showed a significant correlation between relative abundance of gut microbial phyla and sedentary lifestyle (r = 0.64, P < 0.0001) as well as metabolic profiles (r = 0.31, P=0.023). ALT: alanine aminotransferase; CCA: canonical correspondence analysis; HDL-C: high density lipoprotein cholesterol; LDL-C: low density lipoprotein cholesterol. [file 3029582.f1.zip › 3029582.f1/3029582supl.docx]
